# Activation of gga-miR-155 by reticuloendotheliosis virus T strain and its contribution to transformation

**DOI:** 10.1099/jgv.0.000718

**Published:** 2017-04-28

**Authors:** Yongxiu Yao, Deepali Vasoya, Lydia Kgosana, Lorraine P. Smith, Yulong Gao, Xiaomei Wang, Mick Watson, Venugopal Nair

**Affiliations:** ^1^​ Avian Viral Disease Programme & UK-China Centre of Excellence on Avian Disease Research, The Pirbright Institute, Pirbright, Ash Road, Guildford, Surrey GU24 0NF, UK; ^2^​ The Roslin Institute and Royal (Dick) School of Veterinary Studies, University of Edinburgh, Easter Bush EH25 9RG, UK; ^3^​ Division of Avian Infectious Diseases, State Key Laboratory of Veterinary Biotechnology, Harbin Veterinary Research Institute, Chinese Academy of Agricultural Sciences, Harbin, PR China

**Keywords:** v-*rel*, NF-κB, miR-155, transformation

## Abstract

The v-*rel* oncoprotein encoded by reticuloendotheliosis virus T strain (Rev-T) is a member of the *rel*/NF-κB family of transcription factors capable of transformation of primary chicken spleen and bone marrow cells. Rapid transformation of avian haematopoietic cells by v-*rel* occurs through a process of deregulation of multiple protein-encoding genes through its direct effect on their promoters. More recently, upregulation of oncogenic miR-155 and its precursor pre-miR-155 was demonstrated in both Rev-T-infected chicken embryo fibroblast cultures and Rev-T-induced B-cell lymphomas. Through electrophoresis mobility shift assay and reporter analysis on the gga-miR-155 promoter, we showed that the v-*rel*-induced miR-155 overexpression occurred by the direct binding to one of the putative NF-κB binding sites. Using the v-*rel*-induced transformation model on chicken embryonic splenocyte cultures, we could demonstrate a dynamic increase in miR-155 levels during the transformation. Transcriptome profiles of lymphoid cells transformed by v-*rel* showed upregulation of miR-155 accompanied by downregulation of a number of putative miR-155 targets such as Pu.1 and CEBPβ. We also showed that v-*rel* could rescue the suppression of miR-155 expression observed in Marek’s disease virus (MDV)-transformed cell lines, where its functional viral homologue MDV-miR-M4 is overexpressed. Demonstration of gene expression changes affecting major molecular pathways, including organismal injury and cancer in avian macrophages transfected with synthetic mature miR-155, underlines its potential direct role in transformation. Our study suggests that v-*rel*-induced transformation involves a complex set of events mediated by the direct activation of NF-κB targets, together with inhibitory effects on microRNA targets.

## Abbreviations

ALV, avian leukosis virus; CEF, chicken embryo fibroblast; DPI, days post-infection; EBV, Epstein–Barr virus; EMSA, electrophoresis mobility shift assay; GST, glutathione S-transferase; MDV, Marek's disease virus; miRNA, microRNA; REV, reticuloendotheliosis virus; Rev-T, reticuloendotheliosis virus T strain.

## Introduction

The *rel*/NF-κB family of transcription factors [1, 2] plays a key role in the control of cell proliferation and apoptosis, two functions critical in cancer. The involvement of *rel*/NF-κB in malignancy is best demonstrated by the acute oncogenicity of their viral derivative, v-*rel*, first identified in reticuloendotheliosis virus T strain (Rev-T) [3, 4]. Rev-T is an acutely transforming variant of reticuloendotheliosis virus (REV), the aetiological agent of reticuloendotheliosis in birds, carrying the viral oncogene v-*rel*, a variant of the turkey cellular proto-oncogene c-*rel* [5–7]. Because of the rapidity and efficiency of transformation of the cells, v-*rel* provides a valuable model for studying the role of the *rel*/NF-κB family in neoplastic transformation and cancer. The v-*rel*-mediated transformation occurs predominantly through the modulation of transcription of *rel*/NF-κB targets [8–10], examples of which include AP-1 [11, 12], IRF-4, [13] SH3BGRL [14], TGFβ/Smad [15] and telomerase reverse transcriptase (TERT) subunit [16]. More recently, repression of BLNK and BCAP proteins [17] and a novel interaction of CAPERα and the transactivating domain of v-*rel* [18] were shown to be important for lymphocyte transformation by the v-*rel* oncoprotein.

Several studies have also implicated microRNAs (miRNAs) as key mediators of a number of cell regulatory processes including the induction of cancer [19–21]. Among the numerous miRNAs expressed in haematopoietic cells, miR-155 was shown to have the most wide-ranging effects on the biology of lymphocytes [22–25]. It is processed from a primary transcript, known as ‘*Bic*’ (B-cell integration cluster), whose upstream region was originally found to be a frequent site of integration of the avian leukosis virus (ALV) in lymphomas [26]. A number of recent miRNA profiling studies have shown elevated levels of miR-155 in a wide array of cancers including lymphomas [27–30].

In a recent study on chicken embryo fibroblast (CEF) cultures infected with reticuloendotheliosis virus HA1101 strain, differential expression of a number of genes leading to changes in several signalling pathways was reported [31]. We and others have shown upregulation of miR-155 in Rev-T-transformed cell lines and CEFs [32, 33]. For further analysis of the global changes in miRNA profiles induced by v-*rel*, we used an *in vitro* model of v-*rel*-induced transformation of embryonic splenocytes to demonstrate the sequential upregulation of miR-155 during the transformation process. Our studies confirm that v-*rel*-mediated upregulation of gga-miR-155 occurs through the direct binding to at least one of the putative NF-κB sites on the *Bic*/miR-155 promoter. Analysis of the gene expression changes in the v-*rel*-transformed cells further demonstrated downregulation of a number of known miR-155 targets potentially affecting a number of important biological pathways. Demonstration of the targeting of a number of cancer-related genes in chicken macrophages overexpressing miR-155 demonstrated the importance of this miRNA as a major regulator of v-*rel*-induced transformation.

## Results

### Upregulation of miR-155 in Rev-T-transformed cell lines

**Fig. 1.**
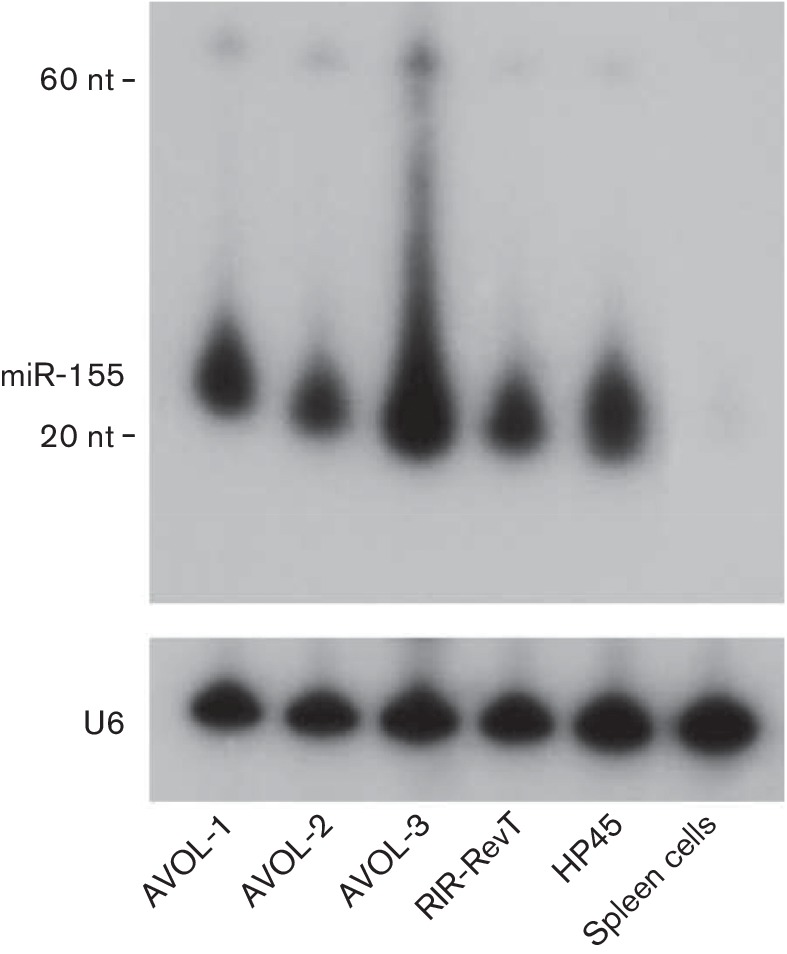
Northern blotting analysis for determining miR-155 expression. Twenty micrograms of total RNA extracted from the cells indicated was separated on a 15 % denaturing polyacrylamide gel, blotted and hybridized with end-labelled antisense oligonucleotide probes to gga-miR-155. Size markers to indicate the positions of the pre-miRNA and mature miRNA are shown. The cellular U6 small nuclear RNA served as a loading control.

During the analysis of the global changes in miRNA expression in chicken lymphocyte lines transformed by avian oncogenic viruses, we observed that miR-155 was overexpressed in v-*rel*-transformed chicken lymphocytes, compared with the normal spleen cells and Marek’s disease virus (MDV)-transformed cell lines [32]. For confirmation of the higher expression of miR-155 in v-*rel*-transformed cells, we examined the Rev-T-transformed cell lines AVOL-1, AVOL-2, AVOL-3 and RIR-Rev-T by Northern blot analysis. An ALV-transformed B-cell line, HP45, was used as a positive control where miR-155 is upregulated due to insertional activation, and normal spleen cells, which do not express detectable levels of miR-155, were used as a negative control. High levels of miR-155 transcripts were readily observed in all Rev-T transformed cell lines (Fig. 1).

### v-*rel* binds to the NF-κB sites in the Bic/miR-155 promoter

**Fig. 2.**
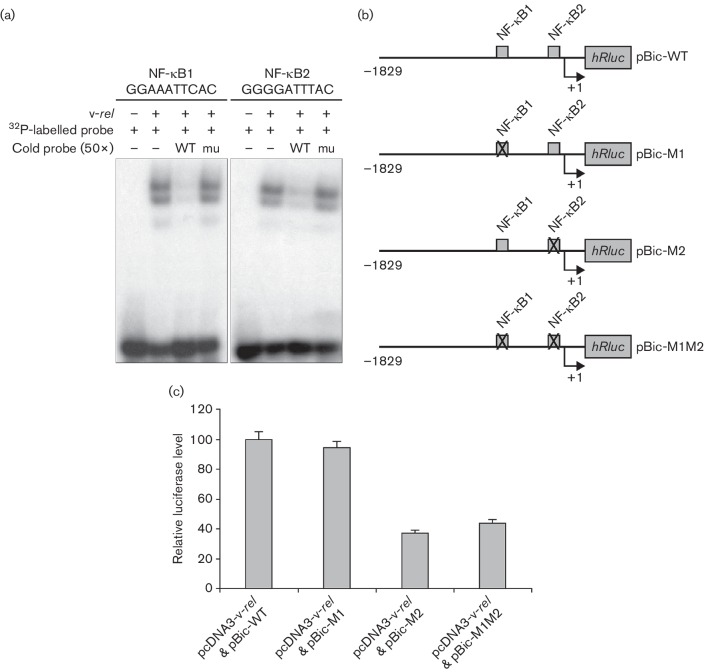
Activation of miR-155 by v-*rel* occurs through the NF-κB pathway. (a) Electrophoresis mobility shift assay using purified v-*rel* on the two putative NF-κB binding sites, NF-κB1 (**−**581) and NF-κB2 (−66), on the chicken *Bic*/miR-155 promoter. WT, 50-fold molar cold wild-type competitor; mu, 50-fold molar cold mutant competitor. (b) Schematic diagram of luciferase reporter constructs carrying the wild-type (WT) and mutant (M1, M2 and M1M2) chicken *Bic*/miR-155 promoter. (c) Relative levels of luciferase in DF-1 cells co-transfected with pcDNA3-v-*rel* and the reporter constructs. Error bars represent the data from four replicates.

Having demonstrated the upregulation of miR-155 in Rev-T transformed cells, we examined the potential mechanisms of miR-155 overexpression by v-*rel*. Analysis of the chicken *Bic*/miR-155 promoter sequence for potential transcription factor binding sites using the program tfsearch [34] identified a number of transcription factor binding sites, including two putative NF-κB sites (NF-κB1 and NF-κB2) located at positions −581 and −66, respectively (relative to the transcription start site). In order to establish that v-*rel* binds directly to the putative NF-κB sites in the *Bic*/miR-155 promoter, an electrophoresis mobility shift assay (EMSA) was carried out using a recombinant glutathione S-transferase (GST)–v-*rel* fusion protein. Briefly, purified GST-v-*rel* protein was incubated with a dsDNA oligonucleotide probe spanning the two putative NF-κB sites. Intense shifted bands were observed with incubation of GST-*v-rel* and wild-type labelled probes for both sites (lane 2, Fig. 2a). The bands were competed by an excess of cold competitor (lane 3, Fig. 2a), but not by the same amount of a mutant competitor that was not bound by v-*rel* protein (lane 4, Fig. 2a).

### NF-κB site 2 in the *Bic*/miR-155 promoter is required for miR-155 activation

Having demonstrated the direct binding of v-*rel* to the NF-κB sites, we next examined the possible contribution of these elements in mediating *Bic* regulation. To this end, we carried out reporter assays to examine the ability of v-*rel* to drive the expression of the *R* *enilla* luciferase reporter gene using constructs containing the wild-type or the mutant chicken *Bic*/miR-155 promoter. For this, the chicken *Bic*/miR-155 promoter region extending from −1829 to +3 nucleotides from the transcription start site (+1) was cloned upstream of the *Renilla* luciferase gene of the psiCHECK−2 vector (Promega) to replace the SV40 promoter, generating the reporter construct pBic-WT. Mutagenesis of the two NF-κB sites was carried out by overlapping PCR generating the pBic-M1, pBic-M2 and pBic-M1M2 constructs, where the NF-κB1, NF-κB2 or both sites, respectively, were mutated (Fig. 2b). For the reporter assay, each of the reporter and pcDNA3-v-*rel* constructs were co-transfected into DF-1 cells, and the luciferase expression was assayed 48 h later using the Dual-Glo Luciferase Assay System (Promega) following manufacturer’s instructions. As shown in Fig. 2(c), mutation of the first NF-κB site (pBic-M1) did not reveal obvious changes in the luciferase levels compared with the wild-type promoter (pBic-WT) construct. In contrast, mutation of the second NF-κB site (pBic-M2) decreased the promoter activity by 63 % compared with that of the pBic-WT, suggesting that the v-*rel*-mediated transactivation occurs mainly through this NF-κB site. The promoter activity of the double mutant pBic-M1M2 construct was similar to that of the pBic-M2 construct, further confirming that the second NF-κB site in the *Bic*/miR-155 promoter is important for the v-*rel*-mediated upregulation of miR-155.

### v-*rel* relieves the inhibition of miR-155 expression in MSB-1 cells

**Fig. 3.**
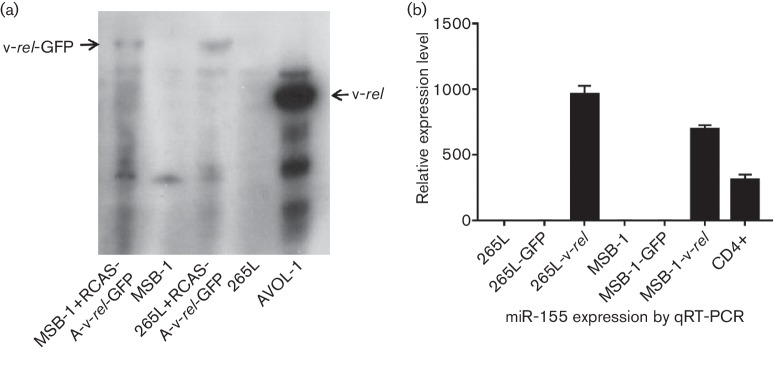
Upregulation of miR-155 in MDV-transformed cell lines by v-*rel*. (a) Cell lysates from MSB-1 and 265L cells infected with RCAS(A)-v-*rel*-GFP were analysed by Western blot using HY87 antibody for v-*rel* expression. Uninfected MSB-1 and 265L cells were included as a negative control, and AVOL-1 cells were included as a positive control. (b) Expression levels of miR-155 in RCAS(A)-v-*rel*-GFP-infected and -uninfected MSB-1 and 265L cells. RCAS(A)-GFP-infected cells were also included as a control.

We have previously shown that miR-155 is consistently downregulated in MDV-transformed tumours and cell lines [32]. Although the mechanisms for this downregulation are not known, this could be due to the complementation of miR-155 functions by the high levels of the viral homologue MDV-miR-M4 expressed in these cells. We wanted to examine whether the downregulation of miR-155 in MDV-transformed cell lines could be rescued by expressing v-*rel* in these cells. RCAS(A)-v-rel-GFP virus stocks were used for transduction of v-*rel* into MSB-1 and 265L cell lines, where the GFP marker allowed sorting of the infected cells. Analysis of the sorted cells by Western blotting showed expression of v-*rel*-GFP in infected MSB-1 and 265L cells but not in uninfected cells (Fig. 3a). Expression of v-*rel* increased the level of miR-155 expression by approximately 700-fold in MSB-1 cells and by about 900-fold in 265L cells, which is much higher than the miR-155 level in non-transformed CD4^+^ cells (Fig. 3b), demonstrating that ectopic expression of v-*rel* can induce expression of miR-155 in avian lymphoid cells.

### Induction of miR-155 is accompanied by downregulation of potential targets

**Fig. 4.**
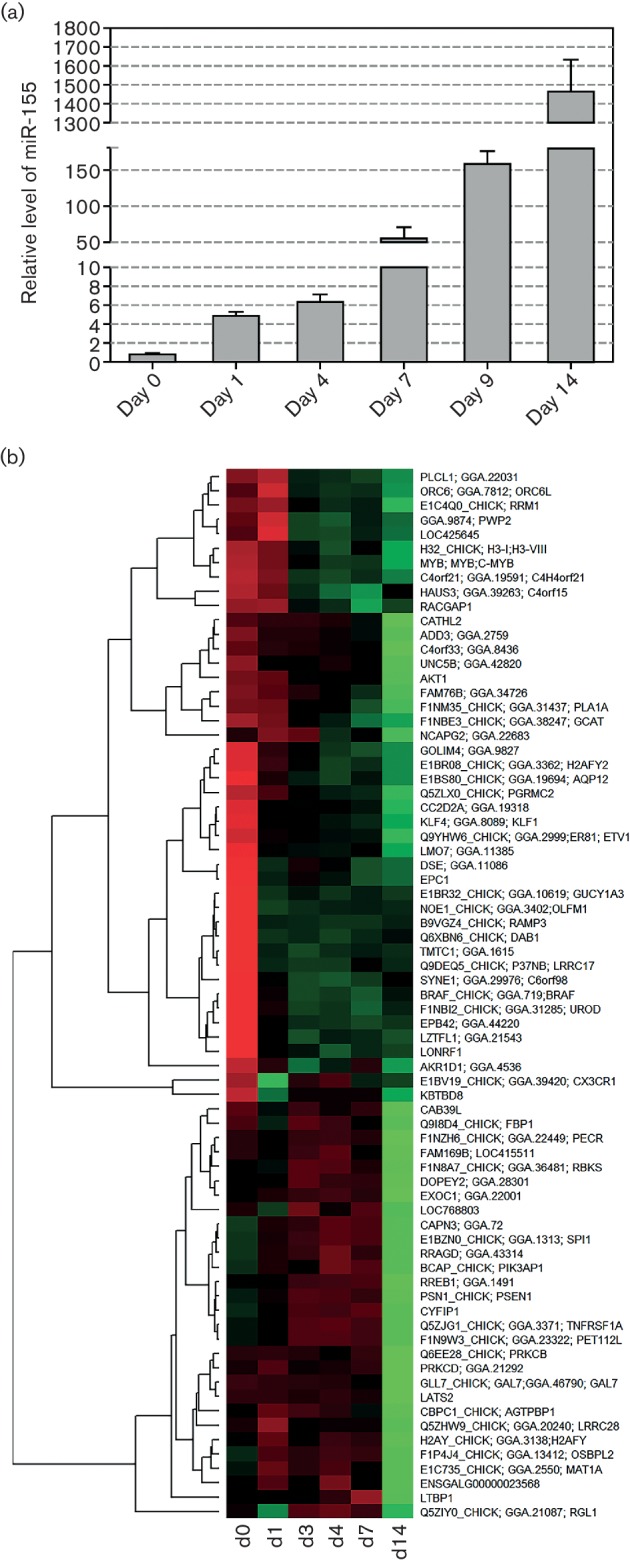
Upregulation of miR-155 during v-*rel* transformation is associated with downregulation of targets. (a) Expression levels of miR-155 in RCAS(A)-v-*rel-*transformed embryonic splenocytes measured in RNA samples harvested on days 0, 1, 4, 7, 9 and 14 post-infection. (b) Heat map of 73 downregulated genes predicted to be targets of gga-miR-155. Affymetrix probes were analysed using Limma, comparing d14 to d0, and those with an FDR ≤0.01 and fold-change ≤−1 (twofold) were selected. The list was further filtered for those genes predicted to be targeted by gga-miR-155. The heat map was drawn in R using the Pearson correlation coefficient as a distance measure.

For further analysis of the dynamic global changes in miRNA profiles during v-*rel*-induced transformation, we examined the changes in RCAS(A)-v-*rel*-infected chicken embryonic splenocytes undergoing transformation. Induction of v-*rel* in these cells resulted in rapid transformation resulting in the appearance of continuously proliferating cell lines usually in 8–10 days. The dynamic changes of miR-155 expression during the transformation process of splenocytes measured by qRT-PCR are shown in Fig. 4(a). Quite clearly, miR-155 is significantly upregulated during the time-course of v-*rel* transformation, with levels showing increases of fivefold (day 1), sixfold (day 4), 50-fold at day 7, 150-fold at day 9 and nearly 1500-fold at day 14, as compared with the level at day 0.

**Fig. 5.**
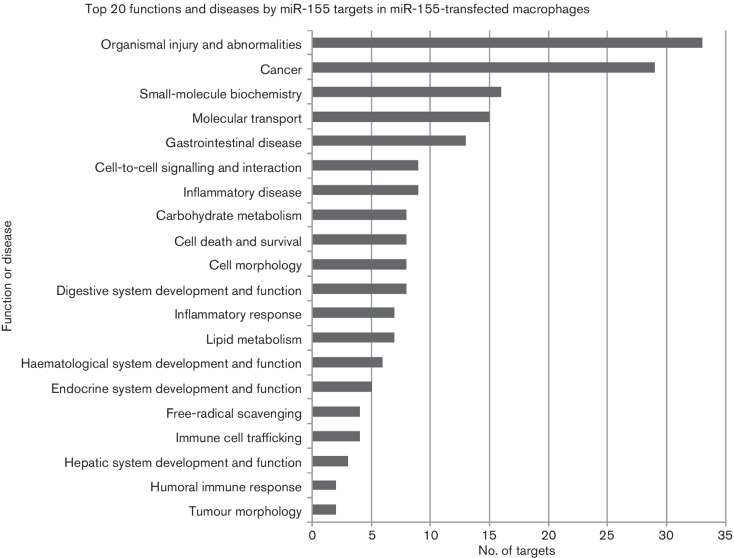
Potential miR-155 targets are involved in a number of diseases and functions. Top 20 functions (sorted by *P* value) of the miR-155 targets identified in primary avian macrophages transfected with miR-155 mimics. Grey bars indicate the number of potential target genes for each disease or function.

In order to assess the simultaneous changes in gene expression during transformation, we carried out a transcriptome analysis using the chicken Affymetrix platform using the RNA samples extracted from these cells. To focus on miRNA-induced repression of gene expression, we used the Bioconductor package Limma [35] to extract 1242 genes that showed significant downregulation at day 14 compared with day 0. Table 1 shows the top 20 statistically enriched predicted miRNA targets in this list. Of the 1242 downregulated genes, 73 are predicted targets of gga-miR-155 (Fig. 4b), making it the top hit of the most enriched miRNA targets. Analysis also showed that the enrichment of the targets of other miRNAs such as gga-miR-9*, gga-miR-217, gga-miR-19a and gga-miR-23b was also significant. These data highlighted the importance of miR-155 and other miRNAs in *v-rel*-induced transformation. MiR-155 is a well-studied oncogene of haematopoietic cells. Considering the complexity of target analysis in the v-*rel*-induced transformation system, as many miRNAs and mRNAs are affected by v-*rel*, we overexpressed miR-155 in chicken macrophages derived from line 0 chicken by transfection of miR-155 mimics into bone-marrow-derived macrophages. ‘Allstars’ negative control (Qiagen) was used as control in an attempt to get a cleaner result on miR-155 targets. The RNA extracted from transfected cells was analysed by deep sequencing. The significantly downregulated genes with miR-155 target sites in the 3′ UTR were subjected to pathway analysis using the Ingenuity Pathway Analysis tool. As shown in Fig. 5, several potential miR-155 targets are involved in a number of diseases and cellular processes. The number of cancer-related genes targeted by miR-155 ranks second, implicating the importance of miR-155 as a regulator in disease pathogenesis, particularly in tumorigenesis.

**Table 1.** Top 20 enriched miRNA targets in the list of 1242 downregulated genes

microRNA	Number of miRNA target genes in the population			FDR||
	Predicted†	Expected‡	Observed§	
gga-mir-155	581	45	73	0.002¶
gga-mir-9*	504	39	65	0.002¶
gga-mir-217	603	46	69	0.033¶
gga-mir-19a	648	50	72	0.045¶
gga-mir-23b	633	49	70	0.045¶
gga-mir-106	685	53	74	0.055
gga-mir-137	570	44	63	0.065
gga-mir-20a	727	56	77	0.065
gga-mir-124b	557	43	61	0.065
gga-mir-190	549	42	60	0.069
gga-mir-19b	629	48	67	0.069
gga-let-7k	623	48	66	0.077
gga-mir-466	806	62	82	0.080
gga-mir-17-5p	732	56	75	0.095
gga-mir-302b	652	50	67	0.114
gga-mir-135a	646	50	66	0.115
gga-mir-29b	692	53	70	0.115
gga-mir-124a	577	44	60	0.115
gga-mir-153	621	48	64	0.115
gga-mir-146b*	490	38	24	0.122

†Predicted: the total number of genes predicted to be targets of the microRNA in the population.

‡Expected: the number we would expect to see in our sample by random chance based on our sample size.

§Observed: the number we actually observed.

||FDR: the Benjamini and Hochberg adjusted *P* value from a two-tailed Fisher's exact test.

¶FDR≤0.05.

## Discussion

The Rev-T avian retrovirus encodes the v-*rel* oncoprotein, which is a member of the Rel/NF-κB transcription factor family. Although Rel/NF-κB transcription factors have been associated with oncogenesis in mammals, v-*rel* is the only member of this family that is oncogenic in animal systems. Due to its pervasive role in oncogenesis there is great interest in NF-κB signalling, and v-*rel* provides a valuable model for studying NF-κB signalling in lymphoid cell cancers because of its ability to transform chicken lymphoid cells [12, 15]. In this study, we demonstrate that v-*rel* can readily induce transformation of lymphocyte populations, and the establishment of CD4^+^ T-cell (AVOL-1) and B-cell (AVOL-2) lineages suggested that the v-*rel*-induced transformation function is not restricted to specific lineages.

In addition to the changes in protein-coding genes, many changes in the miRNA profiles also occur in v-*rel*-transformed cells, and one of the miRNAs expressed at significantly higher levels in v-*rel*-derived tumour cell lines such as KBMC and CM758 is gga-miR-155 [33]. Higher expression of miR-155 is reported in a number of haematopoietic malignancies [36–40]. The precursor of miR-155, termed c-*Bic*, was first observed to cooperate with *myc* in chicken B-cell lymphomas induced by avian leukosis proviral integrations [26, 41]. Southern blot hybridization of genomic DNA from AVOL-1 and AVOL-2 cells showed no evidence of genomic rearrangements in *Bic* loci (data not shown) discounting insertional activation of miR-155 in these cell lines. It is known that miR-155 can also be induced by a variety of immune cell stimuli such as TLR ligands, TNF-α, IFN-β and other antigens [41–45]. A conserved AP-1 element in the human *Bic*/miR-155 promoter was shown to be essential for some of these functions [46]. Transcriptional regulation of miR-155 by the TGF-β/Smad4 pathway using the Smad response elements in the human miR-155 promoter has also been reported [47]. Epstein–Barr virus (EBV) latent membrane protein-1 (LMP1) is a potent inducer of miR-155, and the NF-κB sites in the *Bic*/miR-155 promoter have been shown to be pivotal for this function [48, 49].

Both Northern blotting and microarray data showed that miR-155 is significantly increased in v-*rel*-transformed T and B lymphocytes compared with the normal spleen cells. These observations are similar to the findings reported previously [33]. Despite the consistent demonstration of transformation of B and T lymphocytes by v-*rel*, the precise mechanisms have not been demonstrated. As an NF-κB homologue [8], the most likely mechanism of miR-155 upregulation would be through the direct activation of the miR-155 promoter through the NF-κB binding sites. EMSAs showed that v-*rel* binds directly to both NF-κB binding sites. To assess the ability of v-*rel* to activate transcription from the miR-155 promoter, we performed reporter assays using the miR-155 promoter and its derivative lacking each of the NF-κB binding sites. Our results demonstrated that indeed v-*rel* controls miR-155 through one of the NF-κB binding sites in the *Bic*/miR-155 promoter.

A number of previous studies have demonstrated robust expression of *Bic* in EBV-infected cells [50, 51]. It has been shown more recently that EBV-encoded latent membrane protein-1 (LMP-1), a functional homologue of the tumour necrosis factor receptor family, upregulates the expression of miR-155 mainly by activating the NF-κB pathway [48]. The data herein are the first evidence to our knowledge showing miR-155 being regulated by an NF-κB transcription factor, the v-*rel* oncogene encoded by Rev-T in avian systems. It has been shown previously that v-*rel* exerts downstream effects through the transcription factor AP-1 [12, 46]. AP-1 sites are present in chicken *Bic*/miR-155 promoter sequences, and the contribution of AP-1 in regulation of miR-155 expression in v-*rel*-transformed lymphocytes remains to be determined.

Interestingly, while miR-155 was upregulated in Rev-T-transformed cell lines, it was consistently downregulated in MDV-transformed lymphocytes [52]. Although miR-155 functions are probably rescued by the high-level expression of the MDV1-miR-M4 homologue in these cells [53], the precise molecular mechanisms of downregulation of miR-155 in MDV-transformed cells are not clear. RCAS-mediated transduction of v-*rel* did rescue the expression of miR-155 in two of the MDV-transformed cell lines, MSB-1 and 265L. The increased level of miR-155 expression after introduction of v-*rel* into these cells indicated that the upregulation of miR-155 is a direct effect. It is interesting to know that the common occurrence of MDV with REV in chickens could lead to a part or the entire genome of REV integrating into the MDV genome [54, 55]. Although a number of field MDV isolates with REV insertions have been characterized, the precise molecular mechanisms for the altered pathogenic properties and the increased virulence are still not clear [55, 56].

A number of targets of miR-155 have been identified previously. C-Maf [43], AID [57, 58], Pu.1 [59], SOCS1 [60], interleukin-1 [61] and IKKε [49, 62] have been implicated in mediating functions of miR-155 in the immune system. Ets-1 and Meis1 mediate megakaryopoiesis [63]. SHIP1 and C/EBP have been implicated in myeloproliferative disorders [64, 65]; Peli1 controls the generation and function of T-follicular-helper cells through promoting the degradation of the NF-κB family transcription factor c-Rel [66]; tumour protein p53-inducible nuclear protein 1 (Tp53INP1) is involved in pancreatic cancer [67]; and SOCS1 is involved in promoting γ-chain cytokine signalling to ensure effector and memory CD8^+^ T-cell differentiation [68]. Additionally, miR-155 targets JARID2, a cell cycle regulator and part of a histone methyltransferase complex, to promote cell survival [33]. From microarray data on RNA of v-*rel-*transformed cells, 73 out of 1242 significantly downregulated genes are potential targets of miR-155. Not only was miR-155 the most statistically enriched target within the list of significantly downregulated genes, but members of the miR-17-92 cluster are also implicated, a cluster which is known to be involved in cancer [69–72], this further emphasizing the role of oncogenic miRNAs in transformation.

The oncogenic effects of miR-155 are mediated through its target mRNAs. The known miR-155 targets Pu.1 and CEBPβ are present in the downregulated genes from microarray analysis in *v-rel*-transformed cells. Together with the evidence that the potential miR-155 targets in macrophages involved in cancer stand out from those targets related to other diseases and functions, this study demonstrates the important role of miR-155 in *v-rel*-induced transformation. Although the precise roles and molecular pathways of miR-155 in *v-rel*-induced transformation are not fully known, its repressive function on transcriptional factors such as Pu.1 and CEBPβ can have wide-ranging effects on the cellular milieu and the global gene expression profiles seen for lymphocytes. Further studies will be required to ascertain the involvement of Pu.1, CEBPβ and/or other miR-155-regulated transcription factors in the regulation of miR-155-inhibited genes. Similarly, the repression of some of the other target genes is also likely to contribute to the induction of haematopoietic cell malignancy. Although upregulation of miR-155 appears to add complexity to regulation of gene expression in v-*rel*-induced malignant transformation, the downstream network of miR-155 targets, or the importance of those target genes in v-*rel*-induced transformation, could be an interesting area to explore.

## Methods

### Transformed cell lines

Rev-T-transformed cell lines AVOL-1 (CD4^+^ T-cell line) [32], AVOL-2 (B-cell origin), AVOL-3, RIR-RevT (a transformed cell line derived from outbred Rhode Island Red chickens) and ALV HPRS F42 strain transformed B-cell line HP45 [73] were used. The MDV cell lines MSB-1 [74] and 265L [32] were used to study the effects of induction of v-*rel*. All the cell lines were grown at 38.5 °C in 5 % CO_2_ in RPMI 1640 medium containing 10 % FCS, 2 % chicken serum, 10 % tryptose phosphate broth, 0.1 % 2-mercaptoethanol and 1 % sodium pyruvate. The CEF-derived cell line DF-1 was grown using methods described by Himly *et al.* [75].

### Chicken splenocytes, CD4^+^ T cells and magnetic cell sorting

Single-cell suspensions of lymphocytes were prepared from spleen tissues of uninfected birds using Histopaque-1083 (Sigma-Aldrich) density-gradient centrifugation. CD4^+^ T cells were isolated by magnetic cell sorting using mouse anti-chicken CD4 antibodies and goat anti-mouse IgG microbeads (Miltenyi Biotec). After each antibody treatment, cells were washed three times with PBS containing 0.5 % BSA. At each wash, the cell suspension was centrifuged at 450 ***g*** for 10 min. Positively stained cells were sorted through an AutoMACS Pro Separator (Miltenyi Biotec). Purity of the sorted cells was confirmed to be >99 % by flow cytometry after labelling with monoclonal anti-goat/sheep IgG–fluorescein isothiocyanate (Sigma) antibody (data not shown).

### Plasmid constructs

The construct pcDNA3.1-v-*rel* was used for reporter assays. For EMSA, recombinant v-*rel* fused in-frame with GST in pGEX2T (GE Healthcare) vector was used. RCAS(A) retroviral vector (Replication Competent ALV LTR with a Splice acceptor) [76] with v-*rel* cloned into the *Cla*I site was used for *in vitro* transformation of embryonic splenocytes. The orientation of the insert was verified by restriction enzyme digestion and sequencing. An RCAS(A)-EGFP-v-*rel* construct with an C-terminal EGFP tag was used for the expression of v-*rel* in MSB-1 and 265 L cells.

### Cloning and mutagenesis of the *Bic*/miR-155 promoter

The chicken *Bic*/miR-155 promoter region extending from −1829 to +3 nucleotides from the transcription start site (+1) was amplified by PCR from the genomic DNA prepared from CEF. The isolated fragments were digested with *Bgl*II and *Nhe*I and cloned into psiCHECK−2 vector (Promega) cut with *Bgl*II and *Nhe*I to replace the SV40 promoter driving the *Renilla* luciferase gene to generate the pBic-WT reporter construct. Mutagenesis of the two NF-κB sites on the pBic promoter was carried out by overlapping PCR using primers 5′-CCACATATTTCCTTGCTGGCTCGAGACATAAATTTTT CTGAG-3′ and 5′-CTCAGAAAAATTTATGTCTCGAGCCAGCAAGGAAATATGTGG-3′ for NF-κB site 1, and 5′-GAAAAGGAAAGCAGGCTCGAGACTCAAGACGGTT AG-3′ and 5′-CTAACCGTCTTGAGTCTCGAGCCTGCTTTCCTTTTC-3′ for NF-κB site 2. The mutant PCR products were used to replace the corresponding fragment in the pBic-WT vector to generate pBic-M1, pBic-M2 and pBic-M1M2 constructs, where the 1st, 2nd and both NF-κB sites respectively, were replaced. In each case, the *Xho*I restriction site introduced during the replacement of the NF-κB motifs allowed the screening of the constructs by *Xho*I digestion. The sequences of the promoter region of all the constructs were confirmed by sequence analysis.

### Dual luciferase reporter assay

Transfection of DF-1 cells was carried out with Lipofectamine 2000 (Invitrogen) as per the manufacturer's protocols. Approximately 3×10^4^ DF-1 cells were seeded in each well of a 96-well plate. Each of the reporter and pcDNA3-v-*rel* constructs was co-transfected into DF-1 cells, and the luciferase expression was assayed 48 h later using the Dual-Glo Luciferase Assay System (Promega) following the manufacturer’s instructions. The relative expression of *Renilla* luciferase was determined with the normalized levels of firefly luciferase. For each sample, values from four replicates representative of at least two independent experiments were used in the analysis.

### Electrophoresis mobility shift assay (EMSA)

Recombinant full-length v-*rel* from pGEX2t-v-*rel* plasmid in BL21 (DE3) induced with 0.5 mM IPTG for 3 h was purified by Glutathione Sepharose 4 Fast Flow (GE Healthcare) according to the manufacturer’s instructions. EMSAs were performed using a gel shift assay system (Promega) according to the manufacturer’s instructions. Double-stranded synthetic oligonucleotides were radiolabelled using [γ-^32^P]ATP (Amersham) and T4 polynucleotide kinase. For each binding reaction, 3 µg purified protein was incubated with 0.25 µg poly[dI-dC] µl^−1^ containing 50 000 c.p.m. of radiolabelled probes and a 50-fold molar excess of unlabelled competitor oligonucleotide when indicated. DNA-binding reactions were carried out for 30 min at room temperature. Competition experiments were performed by pre-incubation with protein in binding buffer for 10 min, after which labelled probe was added for a further 20 min of incubation at room temperature. The DNA–protein complexes were resolved on 6 % DNA Retardation Gel (Invitrogen) and detected by autoradiography.

### Immunoblotting and Northern blotting

For Western blotting, cells were lysed in protein gel sample buffer (8 M urea, 2 % SDS, 10 mM Tris/HCl pH 6.8, 0.05 % bromophenol blue) and separated on a NuPAGE 4–12 % Bis Tris gel (Invitrogen) and transferred onto nitrocellulose membranes using an iBlot gel transfer system (Invitrogen). Western blotting was performed with c-*rel-* and v-*rel*-specific HY87 mouse monoclonal antibody [77], followed by anti-mouse IgG–peroxidase conjugate (Sigma-Aldrich). Membranes were developed with an ECL Western blotting analysis system (Amersham). For Northern blot analysis, total RNA was extracted from cultured cells with a miRNeasy Mini Kit (Qiagen), and 20 µg total RNA resolved using a 15 % polyacrylamide/1×TBE (Tris/borate/EDTA)/8 M urea gel was blotted to a GeneScreen Plus membrane (Perkin-Elmer). DNA oligonucleotides with sequences complementary to candidate miRNAs, end-labelled with [*γ*-^32^P]ATP (Amersham) using T4 polynucleotide kinase (New England Biolabs), were used as high-specific-activity probes. Hybridization, washing and autoradiography were carried out as previously described [78].

### RCAS virus infection

Virus stocks were generated from DF-1 cells transfected with RCAS(A)-v-*rel* and RCAS(A)-v-*rel*-EGFP constructs approximately 5 days after transfection, when nearly 100 % cells were EGFP-positive in the case of the latter construct. For *in vitro* transformation assay, 1 ml (~10^6^ TCID_50_) of RCAS(A)-v-*rel* virus was used to infect 5×10^6^ embryonic splenocytes and harvested at days 0, 1, 4, 7, 9 and 14 post-infection (DPI) for mRNA microarray analysis and miR-155 quantitation. EGFP-expressing RCAS(A)-v-*rel*-EGFP-infected MSB-1 and 265L cells were also sorted and examined for v-*rel* and miR-155 expression.

### Stem-loop qRT-PCR for miR-155

The expression levels of miR-155 were analysed using the TaqMan MicroRNA Assay System (Applied Biosystems) using 10 ng total RNA as a template for reverse transcription. Each reverse transcription reaction was performed twice independently, and each reaction was tested by PCR in triplicate. All values were normalized to the expression of the endogenous let-7a, and levels were calculated as fold-expression change relative to those from uninfected 265L cells.

### Microarray analysis

Triplicate RNA samples for each of the six time-points (0, 1, 3, 4, 7 and 14 DPI) were analysed using the Affymetrix GeneChip Chicken Genome Array. Expression values were calculated using the Robust Multi-Array Average (RMA) function within the Affy bioconductor package [79]. Affymetrix probes were linked to Ensembl genes using Ensembl (v70), and genes were linked to microRNA predicted targets data from the MicroCosm targets database [80].

For the naive prediction of miRNAs involved in the activation of genes from the mRNA expression data, the following analysis was performed: downregulated probes at 14 DPI compared to 0 DPI were determined using Limma [35], with a FDR ≤0.01 [81] and log fold change ≤−1 (twofold downregulated). Statistical enrichment of miRNA targets within the downregulated gene list was calculated using the corna package [82]. Fisher’s exact test was used to calculate *P* values for statistical enrichment, and adjusted for multiple testing [81]. Heat maps were drawn in R using the Pearson correlation coefficient as a similarity measure [83].

In order to analyse the behaviour of predicted gga-miR-155 targets, expression data from Affymetrix probes representing genes predicted to be targets of gga-miR-155 were extracted and analysed as a set.
